# Editorial: The role of non-coding RNAs in gastrointestinal cancer

**DOI:** 10.3389/fonc.2022.1056897

**Published:** 2022-11-08

**Authors:** Divya P. Kumar, Kanjoormana Aryan Manu, Muzafar Ahmad Macha

**Affiliations:** ^1^ Department of Biochemistry, JSS Medical College, Center of Excellence in Molecular Biology and Regenerative Medicine (CEMR), JSS Academy of Higher Education and Research, Mysore, Karnataka, India; ^2^ Department of Immunology, Amala Cancer Research Centre, Thrissur, Kerala, India; ^3^ Watson-Crick Center for Molecular Medicine, Islamic University of Science and Technology, Awantipora, Jammu & Kashmir, India

**Keywords:** non-coding RNA, gastrointestinal cancer, biomarkers, gene regulation, tumorigenesis

Gastrointestinal (GI) cancer including cancers of the colorectum, stomach, liver, oesophagus, pancreas, and gall bladder is a critical public health concern with high morbidity and mortality rates. According to Globocan 2020, GI malignancies account for 27% of the cancer incidence and 37% of all cancer-related deaths worldwide (1). The molecular pathogenesis of GI cancer is complex and the advancement in recent technology has led to the identification of non-coding RNAs (ncRNAs) that are critically involved in the regulation of cellular processes that promote tumor formation and development (2, 3).

Non-coding RNAs (ncRNAs) are RNA transcripts that do not encode proteins and are divided into two types based on the average length: small non-coding RNAs (sncRNAs, fewer than 200 nucleotides), and long non-coding RNAs (lncRNAs, >200 nucleotides). Small ncRNAs are classified into three types: micro RNA (miRNA), small interfering RNA (siRNA), and piwi-interacting RNA (piRNA). The other types of ncRNAs include promoter-associated transcripts (PATs), enhancer RNA (eRNA), circular RNA (circRNA), small nuclear ribonucleic acid (snRNA), small nucleolar RNA (snoRNA), and tRNA-derived fragments (tRF) (4). The ncRNAs are known to regulate the initiation and progression of various cancers, including GI cancers. The ncRNAs can act as tumor suppressors or oncogenic drivers by regulating processes such as proliferation, invasion, apoptosis, autophagy, and metastasis, thereby promoting malignant transformation and cancer progression (5–8). In addition, ncRNAs also control epigenetic processes and gene expression (9). The ncRNAs released by the cancer cells are used as diagnostic and prognostic markers for GI cancers (10).

This special issue entitled “The Role of non-coding RNAs in Gastrointestinal Cancer” features 4 review articles, 12 original research articles, and a systematic review and meta-analysis that provide insights on the regulatory roles of ncRNAs in GI tumor development and progression. This topic had 60 manuscripts submitted, among which only 17 were accepted for publication.

In the review articles, He et al., describe the use of mRNA and non-coding RNAs as biomarkers for the diagnosis and prognosis of colorectal cancers (CRCs). These non-coding RNAs include miRNA, lncRNA, circRNA, SnoRNA, piRNA, and tRNA. Additionally, Jia et al. explain the clinical significance of ncRNAs in CRC. They summarize the most recent research on IncRNA, miRNA, and circRNAs that act as promoters or tumor suppressors in CRC, aiding in proliferation, apoptosis, invasion, metastasis, autophagy, angiogenesis, and chemo resistance. Yang et al., describe the different types of histone post-transcriptional modifications in gastric cancer (GC) with a special focus on the interaction between ncRNAs (IncRNA, miRNA, and circRNA) and histone acetylation and methylation. The review clearly explains how ncRNA-mediated histone modifications promote tumorigenesis in GC. Furthermore, Aishanjiang et al. discuss the potential role of circRNA as hepatocellular carcinoma (HCC) biomarkers. They also shed light on the challenges, limitations of circRNA research, and their therapeutic potential for the treatment of HCC.

Gao et al., provide experimental evidence of how circRNA protein tyrosine kinase 2 (circPTK2) inhibits cell proliferation, migration, and invasion in GC. CircPTK2 has been demonstrated to decrease tumor growth by specifically targeting the miR-196a-3p/AATK (apoptosis-associated tyrosine kinase) axis, raising the possibility that circPTK2 could be used as a therapeutic target for GC. Huang et al., elucidated that tRNA-derived small RNAs play a role in GC development and discovered that serum tRF-31-U5YKFN8DYDZDD can be used as a potential diagnostic biomarker. Huang et al. identified the biological processes and pathways associated with N6-methyladenosine-related lncRNAs in gastric adenocarcinoma. The study provides important evidence towards the development of predictive biomarkers and immunotherapy for gastric adenocarcinoma. Chen et al. demonstrated the upregulation of miR-199b-5p in GC and its role in promoting proliferation, migration, and metastasis by regulating the expression of hedgehog interacting protein (HHIP). These findings suggest the potential of miR-199b-5p/HHIP pathway axis as a promising therapeutic target for GC. Studies by Zou et al., showed that snRNA host gene 8 (SNHG8) promotes the progression of Epstein-Barr Virus (EBV)-associated GC *via* sponging miR-512-5p and targeting Tripartite Motif Containing 28 (TRIM28). Luo et al., have shown that A-kinase interacting protein 1 (AKIP1) promotes cell invasion and stemness in GC by regulating the HIF-1α and β-catenin pathways under hypoxic conditions. Moreover, Wu et al. identified a network of circRNAs, miRNAs, and immune-related mRNAs that regulate GC while researching the molecular mechanisms of GC at the immunological level. These studies provided evidence that tumor cells and the host immune system interact to regulate the pathogenesis and development of GC.

Du et al. have demonstrated the prognostic value of lncRNA LINC02474 and elucidated its role in regulating apoptosis and metastasis of CRC by suppressing the expression of granzyme B (GZMB). Similarly, Zhang et al., showed that lncRNA cancer susceptibility candidate 11 (CASC11) promote proliferation and migration of CRC cells by adsorbing miR646 and miR-381-3p to upregulate RAB11 family-interacting protein 2 (RAB11FIP2) *via* the PI3K/AKT pathway. These findings strongly suggest the therapeutic potential of CASC11 for CRC. Zhang et al. investigated the role of exosomal miR-15a-5p in the development of HCC. They demonstrated that miR-15a-5p derived from cancer cell exosomes inhibits PD1 expression in CD8+ T cells, thereby suppressing the development of HCC. Like wise, Zhang et al., demonstrated the involvement of miR-20a in the proliferation, invasion, and metastasis of HCC by targeting Enhancer of Zeste Homologue 1 (EZH1). Studies by Huang et al., filled the gap in predicting clinical prognosis based on m6A-related lncRNAs in pancreatic cancer. Furthermore, through a series of experiments, a robust m6A-related lncRNA prognostic model was developed for clinical workers to predict pancreatic ductal adenocarcinoma (PDAC) overall survival. The authors have looked into the potential biological mechanisms and signaling pathways of key m6A-related lncRNAs. They have also found a competitive endogenous RNA (ceRNA) network that connects lncRNAs and m6A-regulators *via* miRNAs.

In the study by Fang et al., the authors have performed a systematic review and meta-analysis of 25 studies that included 1260 patients to explore the prognostic role of microRNA 375, 133, 143, and 145 in esophageal carcinoma. The findings demonstrated a substantial correlation between high expression of miR-375, miR-133, miR-143, and miR-145 and a better prognosis in esophageal cancer.

Taken together, this special issue attempts to explore the diverse functions of ncRNAs in the control of gene expression at the epigenetic, transcriptional, and translational levels. The articles also provide evidence of the role of ncRNAs in the regulation of tumor formation, metastasis, immune response, and treatment resistance in GI cancers such as GC, CRC, HCC, and pancreatic cancer.

## Author contributions

All authors listed have made a substantial, direct, and intellectual contribution to the work and approved it for publication.

## Acknowledgments

We would like to deeply thank all the authors for their scientific contributions to this Research Topic, as well as all of the reviewers for their time, efforts, comments, and constructive criticism in refining the manuscript.

## Conflict of interest

The authors declare that the research was conducted in the absence of any commercial or financial relationships that could be construed as a potential conflict of interest.

## Publisher’s note

All claims expressed in this article are solely those of the authors and do not necessarily represent those of their affiliated organizations, or those of the publisher, the editors and the reviewers. Any product that may be evaluated in this article, or claim that may be made by its manufacturer, is not guaranteed or endorsed by the publisher.
